# Circulating adipocyte fatty acid-binding protein is a predictor of cardiovascular events in patients with stable angina undergoing percutaneous coronary intervention

**DOI:** 10.1186/s12872-017-0691-2

**Published:** 2017-10-10

**Authors:** Wataru Takagi, Toru Miyoshi, Masayuki Doi, Keisuke Okawa, Kazumasa Nosaka, Tomoyuki Nishibe, Naoaki Matsuo, Satoshi Hirohata, Hiroshi Ito

**Affiliations:** 1 Department of Cardiology, Kagawa Prefectural Central Hospital, Takamatsu, Japan; 2 Department of Cardiovascular Medicine, Okayama University Graduate School of Medicine, Dentistry and Pharmaceutical Sciences, 2-5-1 Shikata-cho, Okayama, 700–8558 Japan; 3 Department of Medical Technology, Okayama University Graduate School of Health, Okayama, Japan

**Keywords:** Adipocyte, Fatty acid, Coronary artery disease, Risk factor

## Abstract

**Background:**

Adipocyte fatty acid-binding protein (A-FABP) is expressed in both adipocytes and macrophages. Recent studies have shown that A-FABP is secreted by adipocytes and that the A-FABP concentration is associated with obesity, insulin resistance, and atherosclerosis. We have reported that the coronary atherosclerotic burden is associated with the serum A-FABP concentration. In the present study, we investigated whether the serum A-FABP concentration is associated with prognosis in patients with stable angina pectoris who have undergone percutaneous coronary intervention (PCI).

**Methods:**

This was a prospective single-center trial. In total, 130 patients with stable angina pectoris undergoing their first PCI were enrolled from August 2008 to July 2010 at Kagawa Prefectural Central Hospital. The primary endpoints were cardiovascular death, nonfatal myocardial infarction, nonfatal stroke, revascularization, and hospitalization for heart failure.

**Results:**

During the follow-up (median, 50 months; interquartile range, 23–66 months), 49 cardiovascular events occurred. Kaplan–Meier analysis showed that the cumulative incidence of the primary endpoints in the high A-FABP group (median A-FABP concentration of ≥ 18.6 ng/ml) was greater than that in the low A-FABP group. Cox analysis showed that the A-FABP concentration was an independent predictor of cardiovascular events adjusted for age and the presence of multi-vessel disease (hazard ratio, 1.03; 95% confidence interval, 1.01–1.04; *p* = 0.01).

**Conclusion:**

The serum A-FABP concentration is associated with prognosis in patients with stable angina undergoing PCI, suggesting that the serum A-FABP concentration could be useful for risk assessment of secondary prevention.

**Trial registration:**

UMIN Clinical Trials Registry UMIN000029283 (registration date: September 25, 2017), retrospectively registered.

## Background

Stratification for subsequent cardiovascular events among patients with stable angina pectoris who have undergone percutaneous coronary intervention (PCI) is of considerable interest because of the potential to guide secondary preventive therapies [1, 2].

Adipocyte fatty acid-binding protein (A-FABP), also known as aP2 or FABP-4, is a cytoplasmic protein that is abundantly expressed in mature adipocytes and activated macrophages [3]. Substantial experimental evidence shows that A-FABP plays an important role in metabolic deterioration and the development of atherosclerosis [3–6]. We and other investigators previously reported that a higher circulating A-FABP concentration is an independent risk factor for coronary artery disease [7–9]. Recent studies have shown an association between the circulating A-FABP concentration and future cardiovascular disease in patients with end-stage renal disease [10], prevalent coronary heart disease [11], and acute coronary syndrome [12] as well as in a community-based cohort [13].

The aim of this study was to elucidate the association between the circulating A-FABP concentration and subsequent adverse cardiovascular events in patients with stable angina pectoris who have undergone PCI.

## Methods

### Patients

**Fig. 1:**
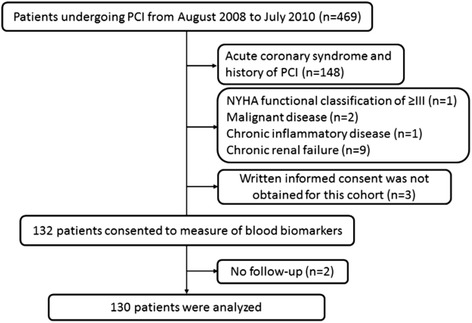
Patients’ enrollment of this study

This was a prospective, single-center trial including patients with stable angina pectoris undergoing their first PCI. Patents’ enrollment was shown in Fig. 1. Four hundred sixty-nine patients underwent PCI from August 2008 to July 2010 at Kagawa Prefectural Central Hospital. Patients were then excluded based on the presence of any of the following criteria: acute coronary syndrome or history of PCI (*n* = 148), New York Heart Association functional classification of ≥ III (*n* = 1), malignant disease with an expected prognosis of <1 year (*n* = 2), chronic inflammatory disease (*n* = 1), and chronic renal failure (serum creatinine concentration of > 2.0 mg/dl) (*n* = 9). As patients who did not provide written informed consent (*n* = 3), 132 patients consented to measurement of blood biomarkers prior to PCI. Of them, patients who had no follow-up data (*n* = 2) were also excluded. Finally, 130 patients were included in the analysis.

This study was approved by the Ethics Committee of Kagawa Prefectural Central Hospital. Written informed consent was provided by all of the patients before the study. The investigation conformed to the principles outlined in the Declaration of Helsinki.

### PCI

PCI was performed using conventional techniques by the femoral or radial approach under systemic heparinization and oral administration of aspirin and clopidogrel. The stent type and inflation pressure were chosen at the discretion of the physicians, who were blinded to the study protocol. Procedural success was defined as reduction of stenosis to < 30% of residual narrowing, improvement of ischemic symptoms, and no major in-hospital complications such as death or emergency bypass surgery. After PCI, the patients received aspirin at 100 mg/day indefinitely and clopidogrel at 75 mg. Revascularization of the original stented target lesion was defined as repeated PCI and was performed in the presence of in-stent restenosis and any symptoms or objective signs of myocardial ischemia.

### Blood sampling

Blood samples were taken after an overnight fast. The serum was separated and stored at −80 °C, and the serum concentration of A-FABP was measured by enzyme-linked immunosorbent assay (BioVendor Laboratory Medicine, Modrice, Czech Republic) [14] during follow-up. With respect to performance, this assay has a < 7% intra-assay and < 5% inter-assay coefficient of variation. Other laboratory parameters were measured using standard laboratory techniques with an automatic analyzer.

### Primary endpoints

The primary endpoints were cardiovascular death, nonfatal myocardial infarction and stroke, revascularization, and hospitalization for heart failure. The time to the first primary endpoint was evaluated retrospectively. All endpoints were defined and assessed according to the statement of the American College of Cardiology/American Heart Association Task Force [15]. Myocardial infarction was defined as type 1 or 2 according to the Third Universal Definition of Myocardial Infarction [16]. Elective coronary revascularization was performed only if the invasive fractional flow reserve of a coronary lesion was ≤ 0.80 [17]. Stroke included both ischemic and hemorrhagic types. Data were carefully collected from the clinical charts and by telephone or letter. Diagnoses was confirmed by an investigator who was blinded to the patients’ data.

### Statistical analysis

Continuous variables are presented as mean ± standard deviation or median (25th, 75th percentiles), and differences between the two groups were evaluated with an unpaired *t*-test or the Mann–Whitney U-test, as appropriate. Categorical variables are presented as number (percentage), and intergroup comparisons were performed using the chi-square test. The Kaplan–Meier analysis of event-free survival during follow-up was based on the median A-FABP concentration. The association between the A-FABP concentration and future events was assessed by Cox proportional hazards analysis. Multivariate Cox proportional hazards analysis was then applied using covariates with *p* value < 0.1 in the univariate Cox proportional hazards analysis. Based on previous studies [11, 12] that reported estimated event rates of 10% and 30% in patients with low and high A-FABP concentrations, respectively, a population of 144 patients would be needed to detect this difference at α = 0.05 and a power of 0.80. A *p* value of < 0.05 was considered statistically significant. Statistical analysis was performed using SPSS 24.0 for Windows (IBM Corp., Armonk, NY).

## Results

Table 1 shows the patients’ baseline characteristics. The patients’ age was 72 ± 9 years, and 81% were male. The proportions of patients with hypertension, dyslipidemia, and diabetes mellitus were 76%, 76%, and 50%, respectively. The patients were divided into two groups according to the median A-FABP concentration (18.6 ng/ml). The high A-FABP group had a higher proportion of men (*p* = 0.01) and a higher body mass index (*p* < 0.01) than the low A-FABP group. There were no significant inter-group differences in age; the presence of hypertension, dyslipidemia, diabetes mellitus, or smoking; or the parameters of lipid and glycemic control. Drug therapies, including administration of antihypertensive drugs and statins, were comparable between the low A-FABP and high A-FABP groups. The distribution of the number of diseased vessels was not significantly different between the two groups.

**Table 1:** Patients’ characteristics

	All (*n* = 130)	low A-FABP < 18.6 ng/ml (*n* = 65)	high A-FABP ≥ 18.6 ng/ml (*n* = 65)	*p* value (high vs. low)
A-FABP (ng/ml)	18.6 (13.8, 27.3)	14.2 (11.2, 16.8)	25.6 (22.6, 34.7)	
Age (years)	72 ± 9	72 ± 9	71 ± 9	0.77
Men, n (%)	105 (81)	47 (72)	89 (81)	0.01
Body mass index (kg/m^2^)	24.5 ± 3.3	23.6 ± 2.8	25.4 ± 3.5	<0.01
Hypertension, n (%)	99 (76)	51 (78)	48 (74)	0.54
Dyslipidemia, n (%)	99 (76)	50 (77)	49 (75)	0.42
Diabetes mellitus, n (%)	65 (50)	33 (51)	32 (49)	0.51
Smoking, n (%)	18 (14)	9 (14)	9 (14)	0.99
LDL cholesterol (mg/dl)	103 ± 28	104 ± 25	102 ± 31	0.64
HDL cholesterol (mg/dl)	43 ± 11	41 ± 10	44 ± 12	0.13
Triglycerides (mg/dl)	141 (102, 181)	132 (97, 187)	148 (118, 178)	0.57
Fasting blood sugar (mg/dl)	99 (92, 119)	99 (92, 119)	100 (92, 120)	0.82
Serum creatinine (mg/l)	0.85 (0.70, 1.00)	0.86 (0.70, 0.96)	0.82 (0.71, 1.01)	0.35
HemoglobinA1c (%)	5.8 (5.3, 6.7)	5.6 (5.2, 6.6)	5.9 (5.3, 6.8)	0.34
hsCRP (mg/l)	0.14 (0.04, 0.36)	0.11 (0.04, 0.34)	0.16 (0.06, 0.41)	0.56
*Number of diseased vessels*				
One	56 (43)	32 (49)	24 (37)	
Two	42 (32)	21 (33)	21 (32)	0.21
Three	32 (25)	12 (18)	20 (31)	
*Medications*				
Antiplatelets	130 (100)	65 (100)	65 (100)	1.00
ACEIs/ARBs	70 (54)	35 (54)	35 (54)	0.99
Calcium channel blockers	64 (49)	33 (51)	31 (48)	0.75
β-blockers	39 (30)	16 (25)	23 (35)	0.18
Statins	75 (58)	40 (62)	35 (54)	0.32

Data are expressed as mean ± standard deviation, or number (%), or median (25th, 75th percentiles)

*LDL* low-density lipoprotein, *HDL* high-density lipoprotein, *A-FABP* adipocyte fatty acid-binding protein, *hsCRP* high-sensitivity C-reactive protein, *ACEI* angiotensin-converting enzyme inhibitor; ARB, angiotensin II receptor blocker

**Fig. 2:**
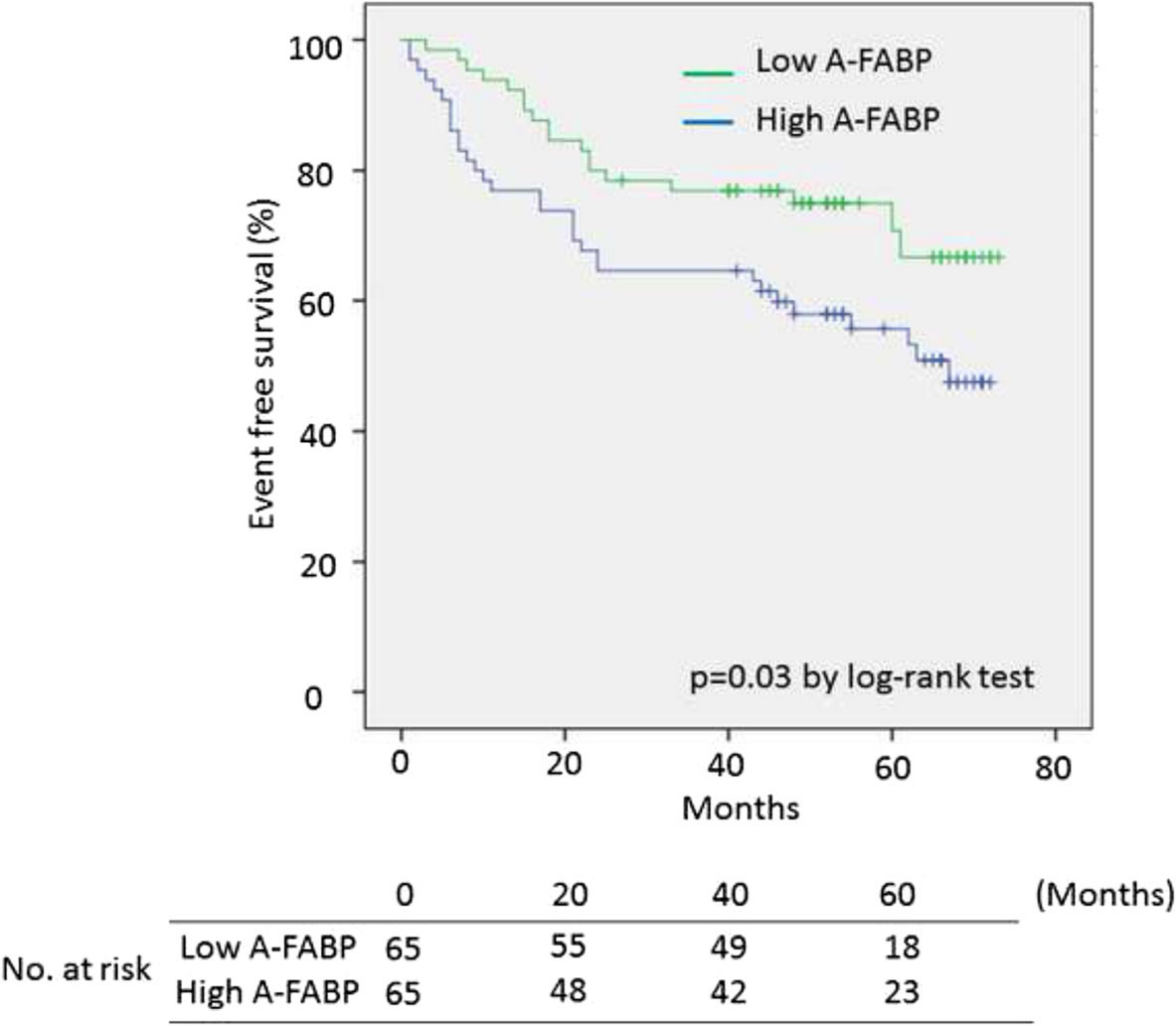
Kaplan–Meier curves for the primary outcomes. Shown are the cumulative event-free rates for the primary endpoint of death from cardiovascular disease, nonfatal myocardial infarction, nonfatal stroke, revascularization, and hospitalization for heart failure (beginning from the time of percutaneous coronary intervention to the day of the first occurrence of a primary endpoint event, the day of the last office or phone visit, or the day of death during follow-up)

During the median follow-up period of 50 months (interquartile range, 23–66 months), we confirmed 49 cardiovascular events (18 in the low A-FABP group and 31 in the high A-FABP group), including 16 cardiovascular deaths (5 in the low A-FABP group and 11 in the high A-FABP group), 6 strokes (4 in the low A-FABP group and 2 in the high A-FABP group), 18 revascularizations (7 in the low A-FABP group and 11 in the high A-FABP group), and 9 hospitalizations for heart failure (2 in the low A-FABP group and 9 in the high A-FABP group). The follow-up durations in the low and high A-FABP groups did not differ significantly (median [interquartile range]: 50 months [36–75 months] and 48 months [18–66 months], respectively; *p* = 0.54). As shown in Table 2, the estimated total event rate in the high A-FABP group was significantly greater at 1 year than that in the low A-FABP group. Kaplan–Meier survival curves showed that the high A-FABP group had a significantly higher mortality rate than the low A-FABP group (Fig. 2). The Cox proportional hazard regression analysis to predict cardiovascular events included A-FABP concentration, age, men, hypertension, diabetes mellitus, dyslipidemia, smoking, multi-vessel disease, and medications (Table 3). The model demonstrated that the A-FABP concentration was a significant explanatory variable after adjustment for the other parameters, suggesting that a higher A-FABP level is an independent predictor of long-term cardiovascular events (hazard ratio, 1.03; 95% confidence interval, 1.01–1.04; *p* = 0.01).

**Table 2:** Primary endpoints at 1 and 3 years

	low A-FABP	high A-FABP	*p* value^a^ (high vs. low)
At 1 year			
Total events	4 (6%)	15 (23%)	0.006
Cardiovascular death	0 (0%)	4 (7%)	0.035
Nonfatal myocardial infarction	0 (0%)	0 (0%)	N/A
Nonfatal stroke	0 (0%)	1 (1%)	0.317
Revascularization	2 (3%)	7 (12%)	0.063
Hospitalization for heart failure	2 (3%)	3 (5%)	0.596
At 3 years			
Total events	15 (23%)	23 (35%)	0.084
Cardiovascular death	4 (7%)	7 (13%)	0.249
Nonfatal myocardial infarction			
Nonfatal stroke	3 (5%)	2 (4%)	0.765
Revascularization	6 (10%)	9 (15%)	0.288
Hospitalization for heart failure	2 (3%)	5 (9%)	0.204

Data are expressed as number and Kaplan–Meier estimated event rates at 1 year and 3 years

^a^Log-rank test was performed for comparison between two groups

*A-FABP* adipocyte fatty acid-binding protein

**Table 3:** Multivariate analysis

	Univariate		Multivariate	
	Hazard ratio (95%CI)	*p* value	Hazard ratio (95%CI)	*p* value
A-FABP, per 1 ng/ml	1.02 (1.01–1.03)	< 0.01	1.03 (1.01–1.04)	0.01
Age, per 1 year	1.04 (1.01–1.07)	< 0.01	1.04 (1.01–1.08)	0.01
Men	1.41 (0.56–2.36)	0.72	–	
Hypertension	1.47 (0.71–3.04)	0.29	–	
Diabetes mellitus	1.01 (0.58–1.77)	0.97	–	
Dyslipidemia	0.86 (0.46–1.62)	0.64	–	
Smoking	0.99 (0.44–2.20)	0.98	–	
Multivessel disease	1.71 (0.95–3.08)	0.08	1.71 (0.95–3.10)	0.08
CCBs	1.09 (0.63–1.92)	0.75	–	
ACEIs/ARBs	1.54 (0.87–2.72)	0.14	–	
Statins	1.25 (0.62–2.52)	0.53	–	
Hypoglycemic agents	0.63 (0.32–1.23)	0.18	–	

*CI* confidence interval, *A-FABP* adipocyte fatty acid-binding protein, *CCBs* calcium channel blockers, *ACEI* angiotensin-converting enzyme inhibitor, *ARB* angiotensin II receptor blocker. Multivariate Cox proportional hazards analysis was then applied using covariates with p value < 0.1 in the univariate Cox proportional hazards analysis

## Discussion

We found that the circulating A-FABP concentration predicts cardiovascular events in patients with stable angina undergoing PCI. Further analysis using a multivariable-adjusted model supported the presence of an independent association between the A-FABP concentration and subsequent cardiovascular events. To our knowledge, this is the first long-term prospective study to demonstrate that the circulating A-FABP concentration is linked to clinical cardiovascular outcomes in patients with stable angina undergoing PCI.

Several studies have evaluated the association between the circulating A-FABP concentration and future cardiovascular events. Chow et al. reported that the circulating A-FABP concentration predicted the development of cardiovascular disease in a community-based cohort [13]. Eynatten et al. identified an association between the circulating A-FABP concentration and long-term prognosis in patients with coronary heart disease [11]. In their study, many participants were patients with an old myocardial infarction undergoing coronary artery bypass grafting, PCI, and noninvasive treatment. Other groups reported the usefulness of the circulating A-FABP concentration as a prognostic biomarker in patients with acute coronary syndrome as well as in those with end-stage renal disease [12, 10]. Our study included patients with stable angina pectoris who underwent PCI, which is an advantage in terms of understanding the clinical relevance of the circulating A-FABP concentration in this specific population.

The present study showed that women had higher A-FABP concentrations than men. Sex-related differences in the circulating A-FABP concentration have been reported in several previous studies [8, 14]. The expression of A-FABP in subcutaneous adipose was higher than that in visceral adipose tissue [18]. Men generally have more visceral fat, and women have more subcutaneous fat. Furthermore, the A-FABP concentration has been shown to be negatively correlated with the free testosterone concentration in women [19], suggesting that testosterone suppresses the expression of A-FAPB. The differences in the regional fat distribution and sex hormones may explain the sex-related difference.

The underlying mechanisms involved in the association between the circulating A-FABP concentration and future cardiovascular events remain unclear. However, there are several potential explanations. First, A-FABP derived from adipose tissue or activated macrophages may directly influence the vasculature. Previous experimental studies have shown that A-FABP induces smooth muscle cell proliferation and inhibits the expression/activation of endothelial nitric oxide synthase in vascular endothelial cells [20, 21]. Another study showed that A-FABP locally produced by perivascular fat and macrophages in vascular plaques contributes to the development of coronary atherosclerosis [22]. Second, the presence of a high circulating A-FABP concentration is a consequence of accumulation of cardiometabolic risks. Our clinical studies showed that the A-FABP concentration is associated with the body mass index; concentrations of triglycerides, high-density lipoprotein cholesterol, adiponectin, and C-reactive protein; and the homeostasis model assessment–insulin resistance value [8, 14, 23–25]. Thus, the circulating A-FABP concentration is closely associated with obesity, insulin resistance, and type 2 diabetes. However, in the present study, the multivariate analysis revealed that the circulating A-FABP concentration was an independent factor associated with future cardiovascular events. In addition, a genotype–phenotype study showed that carriers of the T-87C polymorphism at the A-FABP locus have a reduced risk for coronary heart disease [26]. Basically, A-FABP works as a chaperon of fatty acids in cells. Thus, interaction of A-FABP and its modulation of cardiovascular risk factors may be another explanation.

We demonstrated the usefulness of the baseline circulating A-FABP concentration as a predictor of future cardiovascular events. Meanwhile, the circulating A-FABP concentration can be modified. We previously reported that an angiotensin II receptor blocker reduced the serum A-FABP concentration in hypertensive patients [23]. Other groups showed that the A-FABP concentration decreased by an omega-3 fatty acid [27], a statin [28], and a dipeptidyl peptidase 4 inhibitor [29] and possibly increased by a sodium-glucose cotransporter 2 inhibitor [30]. Experimental studies have identified several compounds that directly block A-FABP [31–33], but no clinical applications are available. The impact of changes in the circulating A-FABP concentration on cardiovascular events remains unknown. Further studies are required to evaluate the direct impact of changes in the circulating A-FABP concentration on clinical outcomes.

### Limitations

This study has several limitations. First, a small number of patients were enrolled. Second, we included only Japanese patients with stable angina undergoing PCI; therefore, our findings cannot be extrapolated to other ethnic groups or patients without cardiovascular disease. In addition, we excluded patients with acute coronary syndrome, history of PCI, a New York Heart Association functional classification of ≥ III, malignant disease with an expected prognosis of < 1 year, chronic inflammatory disease, and chronic renal failure (serum creatinine concentration of > 2.0 mg/dl). Our findings cannot be applied to all patients with stable angina pectoris undergoing PCI. The results must therefore be interpreted with caution. Third, most of the differences between the two groups occurred in the “soft” endpoints of revascularization and hospitalization for heart failure. Thus, it is possible that A-FABP is more closely associated with symptoms or other laboratory results that would result in more intense treatment approaches rather than disease progression itself. Fourth, our study design precluded the investigation of a direct causal relationship. To determine this causal relationship, long-term interventional studies involving therapeutic agents that reduce A-FABP expression or action such as A-FABP inhibitors are warranted.

## Conclusions

The circulating A-FABP concentration is a predictor of subsequent cardiovascular events in patients with stable angina who have undergone PCI. This finding suggests that the circulating A-FABP concentration has the potential to guide secondary preventive therapies.

## Abbreviations

A-FABP: Adipocyte fatty acid-binding protein
PCI: Percutaneous coronary intervention
